# Role of ID Proteins in BMP4 Inhibition of Profibrotic Effects of TGF-β2 in Human TM Cells

**DOI:** 10.1167/iovs.16-20472

**Published:** 2017-02

**Authors:** Avani A. Mody, Robert J. Wordinger, Abbot F. Clark

**Affiliations:** North Texas Eye Research Institute, University North Texas Health Science Center, Fort Worth, Texas, United States

**Keywords:** TGF-β2, BMP4, ID1, ID3, fibronectin, TM cells

## Abstract

**Purpose:**

Increased expression of TGF-β2 in primary open-angle glaucoma (POAG) aqueous humor (AH) and trabecular meshwork (TM) causes deposition of extracellular matrix (ECM) in the TM and elevated IOP. Bone morphogenetic proteins (BMPs) regulate TGF-β2–induced ECM production. The underlying mechanism for BMP4 inhibition of TGF-β2–induced fibrosis remains undetermined. Bone morphogenic protein 4 induces inhibitor of DNA binding proteins (ID1, ID3), which suppress transcription factor activities to regulate gene expression. Our study will determine whether ID1and ID3 proteins are downstream targets of BMP4, which attenuates TGF-β2 induction of ECM proteins in TM cells.

**Methods:**

Primary human TM cells were treated with BMP4, and ID1 and ID3 mRNA, and protein expression was determined by quantitative PCR (Q-PCR) and Western immunoblotting. Intracellular ID1 and ID3 protein localization was studied by immunocytochemistry. Transformed human TM cells (GTM3 cells) were transfected with ID1 or ID3 expression vectors to determine their potential inhibitory effects on TGF-β2–induced fibronectin and plasminogen activator inhibitor-I (PAI-1) protein expression.

**Results:**

Basal expression of ID1-3 was detected in primary human TM cells. Bone morphogenic protein 4 significantly induced early expression of ID1 and ID3 mRNA (*P* < 0.05) and protein in primary TM cells, and a BMP receptor inhibitor blocked this induction. Overexpression of ID1 and ID3 significantly inhibited TGF-β2–induced expression of fibronectin and PAI-1 in TM cells (*P* < 0.01).

**Conclusions:**

Bone morphogenic protein 4 induced ID1 and ID3 expression suppresses TGF-β2 profibrotic activity in human TM cells. In the future, targeting specific regulators may control the TGF-β2 profibrotic effects on the TM, leading to disease modifying IOP lowering therapies.

Glaucoma is a chronic multifactorial neurodegenerative eye disease, affecting 70 million people worldwide.^1–3^ An early sign of the glaucoma is loss of peripheral vision, and further disease progression leads to permanent vision loss. The major risk factor associated with primary open-angle glaucoma (POAG) is elevated IOP.^4,5^ Increased resistance to aqueous humor (AH) outflow, due to defective trabecular meshwork (TM) function, leads to ocular hypertension.^6–9^ Several investigations suggest that disruption of extracellular matrix (ECM) homeostasis and increased deposition of ECM in the TM are responsible for this elevated IOP. In addition, changes in the TM cytoskeleton and deposition of ECM plaque material increase the stiffness of TM tissue, thereby increasing the AH resistance.^9–12^

While ECM turnover and remodeling maintains normal IOP, growth factors, such as TGF-β isoforms (TGF-β1, TGF-β2) and bone morphogenetic proteins (BMP4, BMP7) have a critical role in maintaining ECM equilibrium.^13^ Numerous studies have shown increased TGF-β2 levels, including the active form, in the AH of POAG patients.^14–16^ Several in vitro studies in cultured TM cells showed that activation of TGF-β2 canonical pathway elevates expression of collagens, fibronectin (FN), actin stress fibers, thrombospondin-1, lysyl oxidase (Lox), transglutaminase, and plasminogen activator inhibitor-1 (PAI-1).^12,17–20^ Transforming growth factor-β2 increases ECM deposition in TM tissues and elevates IOP in ex vivo organ culture and in vivo in rodent eyes.^12,21^ These studies link the increased levels of active TGF-β2 to upregulation of ECM deposition in TM tissue, decreased outflow facility, and increased IOP.

Bone morphogenic proteins, BMP receptors, and BMP antagonists are expressed in TM cells and tissues.^22^ Interestingly, BMPs, especially BMP4 and BMP7, antagonize the TGF-β2 effects in TM cells.^23,24^ Bone morphogenic protein 7 inhibits the fibrotic effects of TGF-β2 in cultured TM cells by inducing I-smad7, an inhibitory smad. Our group has demonstrated that BMP4 blocks TGF-β2–induced ECM production in cultured human TM cells.^23^ However, the signaling mechanism for the BMP4 inhibition of TGF-β2 profibrotic effects remains unknown.

Bone morphogenic protein 4 induces early expression of inhibitor of DNA binding proteins (IDs), dominant negative helix-loop-helix (HLH) proteins, in various cell types and regulates cellular functions, including angiogenesis, neurogenesis, and embryogenesis.^25–28^ Inhibitor of DNA binding proteins have a critical role in regulating tissue specific cell proliferation, differentiation, apoptosis, and fibrotic processes.^29,30^ Inhibitor of DNA binding proteins are distinct from other bHLH transcription factors as they lack a basic amino acid DNA binding domain. There are four evolutionarily conserved family members of IDs (ID1, ID2, ID3 and ID4) that have highly similar HLH domain sequences, which are ubiquitously expressed in mammals. The main differences in ID protein sequences lies outside the HLH domains, and the different functions due to these differences among ID proteins are not completely understood.^31^ The HLH domain in IDs heterodimerize with other transcription factors (especially bHLH group proteins) forming nonfunctional transcription complexes and preventing the complex from binding to DNA.^31,32^ Fibrotic pulmonary, dermal, and corneal diseases studies have shown antifibrotic effects of BMPs via ID proteins suggesting the role of ID as an antifibrotic regulator.^33–35^ In a variety of cells, ID1 and ID3 downregulate extracellular components induced by TGF-β, including FN, PAI-1, collagen, and thrombospondin-1.^34,36–38^ Hence, elucidating the downstream pathway of BMP4 in the TM will give us a better understanding and insight into potential disease modifying IOP therapies.

Therefore, we hypothesized that downstream targets of BMP4, ID1, and ID3 will attenuate the pathogenic effects of TGF-β2 in cultured TM cells. In this study, we demonstrated basal expression of IDs (ID1–ID3) in primary human TM cells. We further showed increased expression and nuclear localization of ID1 and ID3 proteins after BMP4 treatment. We also demonstrated the expression of ID1 and ID3 in primary TM cells is BMP pathway–dependent and the crucial roles of ID1 and ID3 in blocking TGF-β2–induced ECM protein expression in cultured TM cells.

## Methods and Materials

### Cell Culture

Human primary TM cells were isolated and characterized from dissected explants of human TM tissues as described previously.^12,39,40^ Donor eyes were obtained from the Lions Eye Institute for Tissue and Research (Tampa, FL, USA) and were managed according to the tenets of the Declaration of Helsinki for human research. The precharacterized primary human TM cells and the stable transformed cell line GTM3 were grown in Dulbecco's modified Eagle's medium (DMEM; Invitrogen-Gibco, Grand Island, NY, USA) containing 10% fetal bovine serum (FBS; Gibco BRL Life Technologies, Grand Island, NY, USA), L-glutamine (0.292 mg/ml; Gibco BRL Life Technologies), and penicillin (100U/ml)/streptomycin (0.1 mg/ml; Gibco BRL Life Technologies).^40–44^ The primary human TM cell strains used are listed in (Table 1).

**Table 1:**
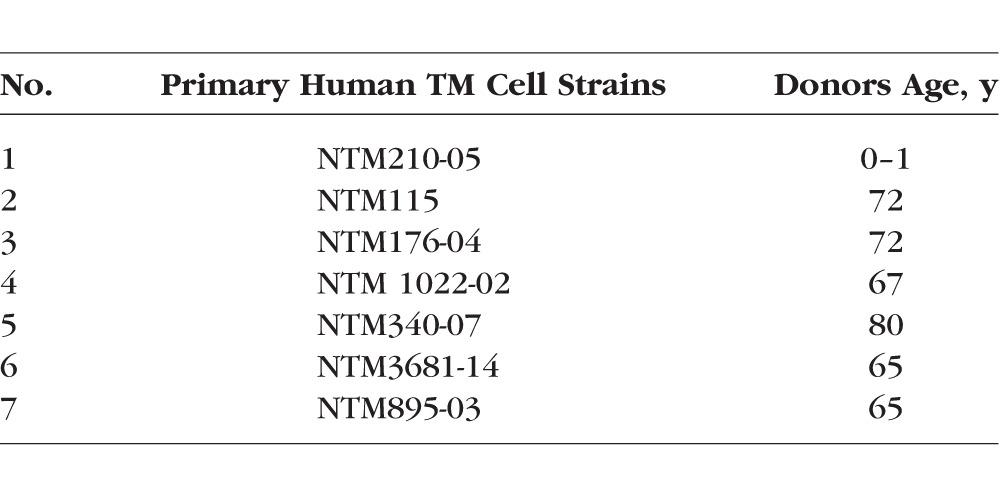
List of Primary Human TM Cells Strains

### TM Cell Treatment

Primary TM cells were grown to 100% confluency. The cells then were serum starved for 24 hours before growth factor or inhibitor treatment. Transformed human TM (GTM3) cells were treated with two different doses of BMP4 (5 and 10 ng/ml; R&D system; Minneapolis, MN, USA) for 12 hours to study dose-dependent mRNA expression of ID1 and ID3. Primary human TM cells were treated with BMP4 (10 ng/ml) for 0 to 48 hours. Cell lysates were collected to study ID1 and ID3 mRNA and protein expression. Primary TM cells were treated with BMPRI inhibitor LDN-193189 (10 and 100 nM; Stemgent, Cambridge, MA) for 6 hours followed by BMP4 (10 ng/ml) for 12 hours of treatment to determine BMP-dependent expression of ID1 and ID3. To test transfection efficiency, ID1 and ID3 expression was studied 48 hours after transfection. To test the effect of ID1 and ID3 on TGF-β2–induced expression of FN and PAI-1, the transfected GTM3 cells were serum starved and treated with TGF-β2 (5 ng/ml) for 48 hours. The experiments in primary human TM cells were repeated at least once in the same cell strain and each cell strain was considered as *n* = 1. In GTM3 cells, experiments were performed in technical replicates (*n* = 3) and each experiment was performed 2 to 3 times.

### Transfection With Expression Plasmids

Plasmid expression vectors for human *ID1* variant1 (pCMV6-XL5-ID1[SC125462]), *ID3* (pCMV6-AC-ID3[SC319486]) and control (pCDNA3.1) were purchased from Origene (Rockville, MD, USA). Trabecular meshwork cell transfection was performed as described in the Origene Protocol for transient transfection of plasmid vectors. In brief, transfection reagent Attractene (Qiagen, Germantown, MD, USA) was used for transfection in serum-free medium (Opti-MEM; Invitrogen). Plasmid vectors mixed with serum-free medium were incubated for 10 minutes. Then plasmid and transfecting reagent were combined and incubated for 20 minutes at room temperature (RT). Transformed human TM (GTM3) cells (1.5 × 10^5^ per ml) were plated into each well of 12 well plates. Cells were then incubated with transfection reagent for 24 hours, washed with PBS, and further incubated in DMEM without serum for the subsequent experiments. Transformed human TM (GTM3) cells were used due to superior transfection efficiency with the plasmid vectors used compared to primary TM cells.

### Reverse Transcription (RT) and Quantitative Real Time PCR (Q-PCR)

Total cellular RNA was extracted from TM cells using Trizol (Invitrogen), and 1 μg of RNA was used for cDNA synthesis. Transcription Super mix (iScript Reverse; Bio-Rad Labs Inc., Richmond, CA, USA) was used for cDNA synthesis. To determine ID mRNA expression, 50 ng/μl of cDNA was used for each reaction. The cDNA was amplified using 10 μl Sso Advance SYBR Super Mix (Bio-Rad Labs) and 100 nM primers sets (Table 2) for each 20 μl of reaction. The RT-PCR products were electrophoresed in a 1.5% agarose gel containing ethidium bromide to detect DNA bands under UV exposure. Quantitative real-time PCR was performed as described previously.^22,43^ The Q-PCR reaction was performed using the Bio-RadCFX96 Real Time system. Each reaction was repeated in triplicates and cycle thresholds (Ct) were normalized to housekeeping gene *GAPDH. GAPDH* was selected as a housekeeping gene since *GAPDH* expression showed no significant change in microarray data obtained from the TM cells treated with BMP4/TGF-β2. The Δ Ct method was used for quantitative analysis. The PCR primers were designed by Prime3 software and were validated by sequencing the PCR product and BLASTing the sequence against the human genome.

**Table 2:**
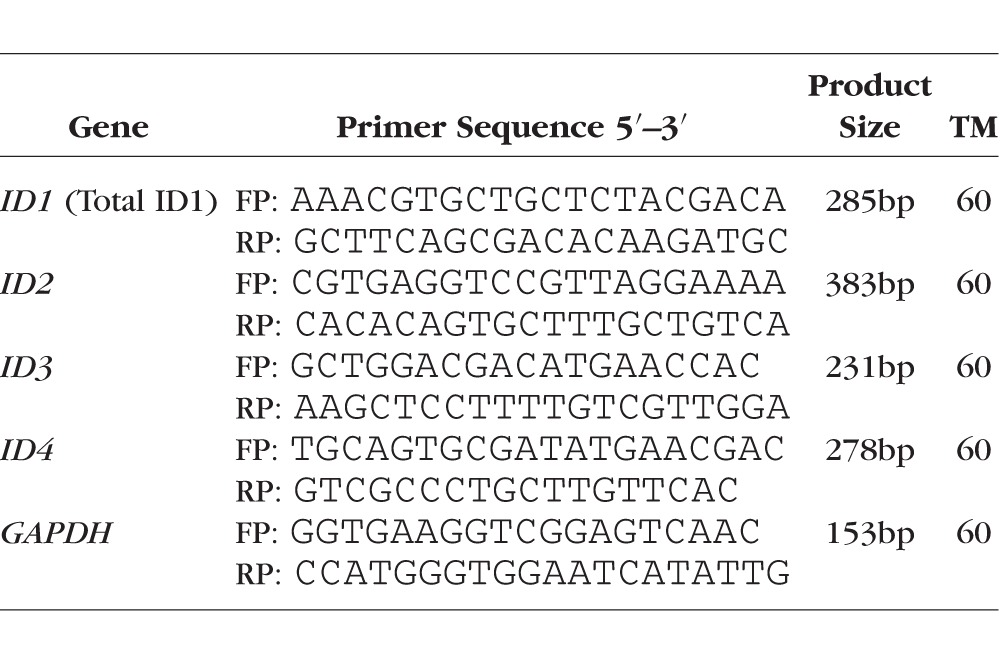
List of PCR Primers

### Protein Extraction and Western Blot Analysis

Total cellular protein was extracted from TM cells using Mammalian Protein Extraction Buffer (Pierce Bio; Thermo Fisher Scientific, Waltham, MA, USA), containing a protease and phosphatase inhibitor cocktail (Pierce Bio; Thermo Fisher Scientific). The Bio-Rad Dc protein assay system (Bio-Rad Labs) was used to determine protein concentrations. The cellular proteins were separated by denaturing 10-15% SDS-PAGE and were electrophoretically transferred to polyvinylidine fluoride (PVDF) membranes. Membranes were blocked in 10% fat-free dry milk in Tris-buffered saline Tween buffer (TBST) for 2 hours at RT. The blocked membranes were incubated overnight with primary antibodies (Table 3) at 4°C. The blots were washed in TBST and incubated with corresponding secondary horseradish peroxidase conjugated antibody diluted (1:10,000) in 5% fat-free milk at RT for an hour. The blots were developed using enhanced chemiluminescence (ECL) detection reagent (Pierce Biotechnology; Thermo Fisher Scientific). The protein images were developed and analyzed using a Fluor ChemTM8900 imager (Alpha Innotech, San Leandro, CA, USA). The same blots were reprobed with an antibody for the housekeeping protein β-actin to ensure equal loading. Beta-actin was selected as loading control since no change was observed in β-actin expression in our microarray data from the TM cells after BMP4/TGF-β2 treatment. For densitometry analysis, the relative density of each protein band was normalized to β-actin. AlphaEaseFC software was used for analyzing images (Alpha Innotech).

**Table 3:**
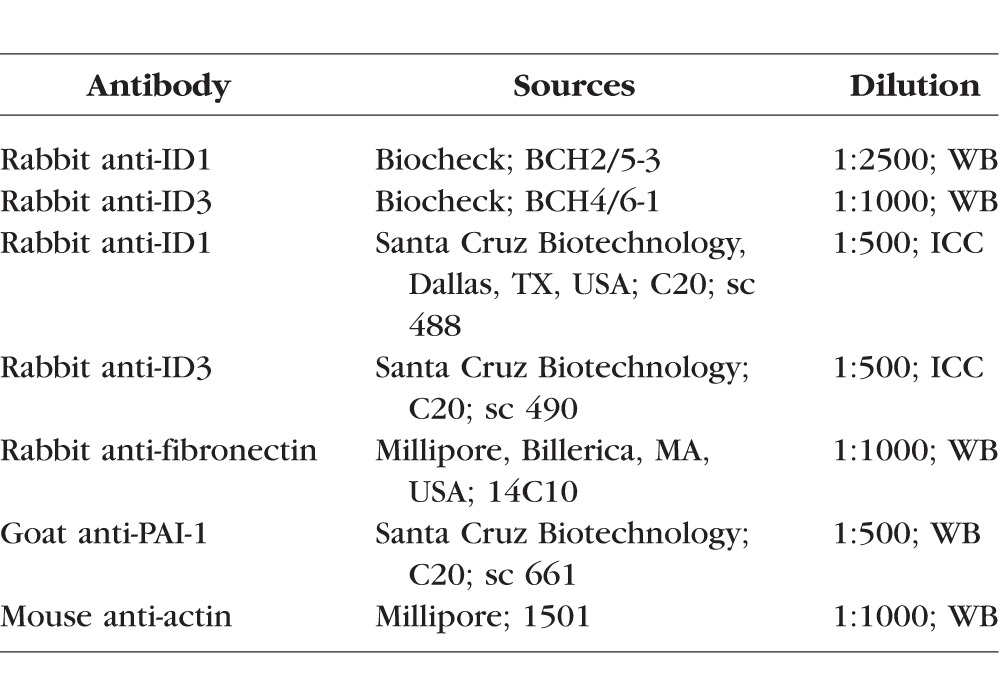
List of Primary Antibodies Used

### Immunocytochemistry

Primary human TM cells (*n* = 4 different strains) were treated with or without BMP4 (10 ng/mL) for 12 hours as reported previously.^45^ In brief, coverslips of each primary human TM cell strain were immunolabeled overnight at 4°C with primary antibodies (Table 3). The primary antibodies used were selected according to previous reports.^26,45,46^ No primary antibody was used as a negative secondary antibody control. Coverslips were incubated for an hour with secondary antibody donkey anti-rabbit-Alexa Fluor 488 (1:1000; Invitrogen), diluted in Triton X-100/PBS. Coverslips were mounted using mounting medium containing 4′,6-diamidino-2-phenylendole (DAPI; Prolong with DAPI; Invitrogen-Molecular Probes) for nuclear staining. Image acquisition was performed using a Nikon Eclipse Ti inverted fluorescence microscope (Nikon, Inc., Melville, NY, USA) equipped with the Cri Nuance FX imaging System (Perkin-Elmer, Inc., Waltham, MA, USA).

### Statistical Analysis

Results between two groups were compared using paired Student's *t*-test. Comparison among 3 or more groups was performed using 1-way ANOVA. The statistical test for each experiment is stated in their respective Figure legend. Statistical analysis was performed using Graph Pad Prism 6 software (Graph Pad Prism, Inc., San Diego, CA, USA). The average value for each group was presented as mean ± SEM, and *P* < 0.05 was considered to be statistically significant.

## Results

### Endogenous Expression of ID mRNA in Human TM Cells

**Figure 1:**
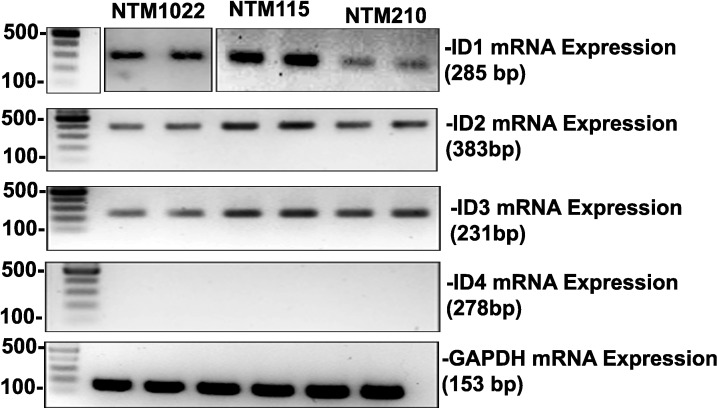
Expression of ID1-4 mRNA in primary human TM cells. RT-PCR was conducted using total RNA extracted from three primary human TM cell strains. Polymerase chain reaction primers are listed in Table 1. The amplified products of ID1, ID2, and ID3 were detected in agarose DNA gels at their expected product size. *GAPDH* was used as internal control for every PCR reaction.

Transcription factors have a key role in maintaining cellular homeostasis and regulatory functions. Inhibitors of DNA binding proteins (ID1–ID4) are essential dominant negative, tissue specific transcription regulators. To study the presence of basal expression of IDs (ID1–ID4), we isolated mRNA from the serum starved normal primary human TM cell cultures (*n* = 3) and performed reverse transcription PCR using ID1 to ID4 primers. We were able to detect basal mRNA expression of ID1, ID2, and ID3 in TM cells (Fig. 1).

### BMP4 Treatment Induced Early ID1 and ID3 mRNA and Protein Expression in Primary Human TM Cells

**Figure 2:**
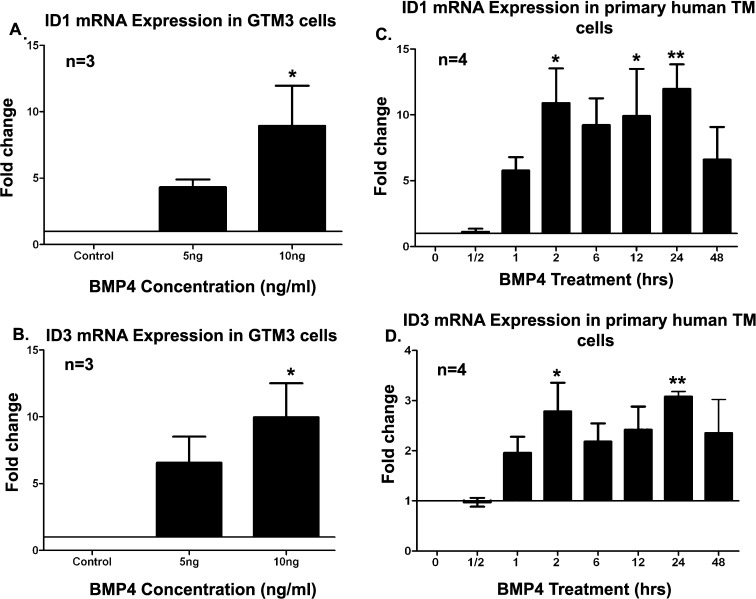
Bone morphogenic protein 4 dose and time-dependent induction of ID1 and ID3 mRNA expression in human TM cells. Transformed human TM (GTM3) cells were treated with two different doses of BMP4 (5 and 10 ng/ml) for 12 hours, and gene expression of ID1 (**A**) and ID3 (**B**) was quantified using Q-PCR. Significant induction of ID1 and ID3 was observed after 10 ng/ml of BMP4 treatment. Following BMP4 (10 ng/ml) treatment from 0 to 48 hours, early induction of ID1 (**C**) mRNA expression was observed within 1 hour with significant fold changes observed at 2, 12, and 24 hours in primary TM cell strains (*n* = 4). Significant ID3 mRNA expression (**D**) was observed at 2 and 24 hours in primary TM cell strains (*n* = 4). Mean ± SEM, * *P* < 0.05, ** *P* < 0.004 when compared by 1-way ANOVA with the Dunnett test. Gene expression of ID1 and ID3 was normalized to *GAPDH* and control group expression (0 hours, no treatment).

**Figure 3:**
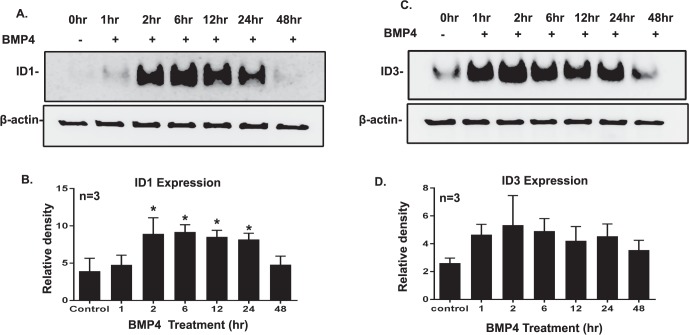
Bone morphogenic protein 4 induced time-dependent expression of ID1 and ID3 proteins in TM cells. Primary human TM cell strains (*n* = 3) were treated with BMP4 (10 ng/ml) for 0 to 48 hours. Protein expression was detected by Western immunoblotting. Western blot images shown represent data for time-dependent induction of ID1 (**A**) and ID3 (**C**) protein expression. Protein expression of ID1 was detected from 2 to 24 hours of BMP4 treatment. Basal expression of ID3 was seen at 0 hours (control), while early induction in ID3 expression after BMP4 treatment was detected from 1 to 48 hours. Beta-actin was used as a loading control. Densitometry analyses of ID1 (**B**) and ID3 (**D**) protein expression. Mean ± SEM, *P < 0.05 when compared by 1-way ANOVA with Dunnett test.

Trabecular meshwork cells and tissues are known to express BMPs (BMP2, BMP4, BMP5, BMP7) and BMP receptors.^22,47^ Bone morphogenic protein 4 binds to BMP-RI/RII and phosphorylates Smads 1/5/8.^23^ While it is well established that ID1 and ID3 are the downstream targets of canonical BMP4 signaling pathway in other tissues, the downstream targets of BMP4 pathway in TM cells remained undetermined. Therefore, we studied the BMP4-induced mRNA of ID1 and ID3 in different primary human TM cell strains. We treated confluent and serum-starved GTM3 cells with different doses of recombinant BMP4 (5 and 10 ng/ml). We observed significant mRNA expression of ID1 and ID3 in TM cells (Figs. 2A, 2B) induced by BMP4 (10 ng/ml) treatment. Further, we treated primary human TM cell strains with BMP4 (10 ng/ml) for 0 to 48 hours to study mRNA and protein expression. We observed early induction of mRNA expression of ID1 and ID3 (*n* = 4) at 1 hour with a significant increase in mRNA expression of ID1 at 2, 12, and 24 hours (Fig. 2C; *P* < 0.05). ID3 mRNA expression significantly increased at 12 and 24 hours of treatment (Fig. 2D; *P* < 0.05). Inhibitor of DNA binding protein 1 protein induction was observed from 2 to 24 hours after BMP4 treatment in 3 different primary human TM cell strains (Fig. 3A). Basal levels of ID3 expression were observed in untreated cells, which increased from 1 to 24 hours after BMP4 treatment (Fig. 3C). Our densitometry analyses of ID1 and ID3 protein expression suggested increased protein expression from basal levels (Figs. 3B, 3D), while the ID1 expression increased significantly from 2 to 24 hours. Hence, our data showed that BMP4 treatment induced ID1 and ID3 mRNA and protein expression in primary human TM cells.

### Exogenous BMP4 Increased the Nuclear Localization of ID1 and ID3 in Primary Human TM Cells

**Figure 4:**
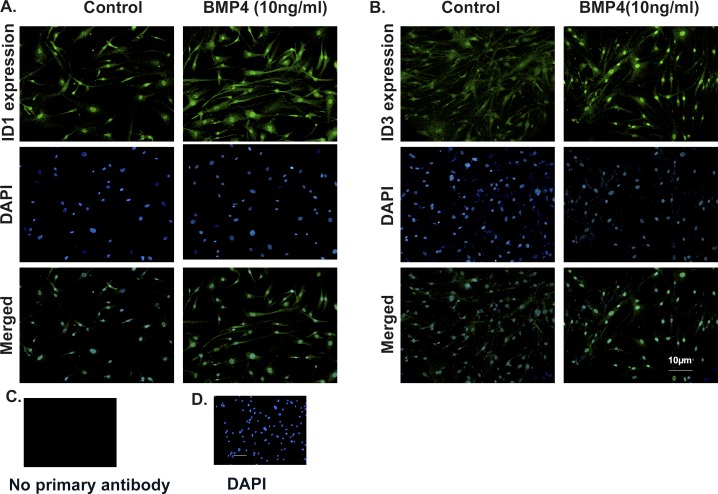
Expression of ID1 and ID3 is increased in the cytoplasm and nucleus after BMP4 treatment in primary human TM cells. Primary human TM cell strains (*n* = 4) were treated with BMP4 (10 ng/ml) for 12 hours. Expression of ID1 (**A**) and ID3 (**B**) protein was compared between control and BMP4 treatment groups by immunocytochemistry (×200). The slides were costained with DAPI to differentiate nuclei in TM cells. Following BMP4 treatment, ID1 cytoplasmic and nuclear expression increased in the TM cells, while ID3 expression and localization increased in nuclei in the TM cells. (**C**, **D**) Primary antibody was omitted as a negative control.

Inhibitors of DNA binding proteins 1 and ID3 are transcription regulator proteins; therefore, the intracellular localization of ID1 and ID3 has an important role in determining their function. Previous studies have shown that ID1 and ID3 are localized in the cytoplasmic and nuclear regions.^48^ We used four different primary human TM cell strains to study ID1 and ID3 protein expression and localization after BMP4 treatment. Our results showed increased ID1 expression in cytoplasm and nucleus (Fig. 4A), while ID3 localized mainly in the nucleus (Fig. 4B) after BMP4 (10 ng/ml) treatment, compared to untreated control cells. There was no ID1 and ID3 immunostaining with omission of the primary antibodies (negative control; Figs. 4C, 4D). The detailed expression of ID1 and ID3 is demonstrated in ×400 images (Supplementary Fig. S2).

### Effect of BMPRI Inhibitor on BMP4 Regulation of ID1 and ID3 in Primary Human TM Cells

**Figure 5:**
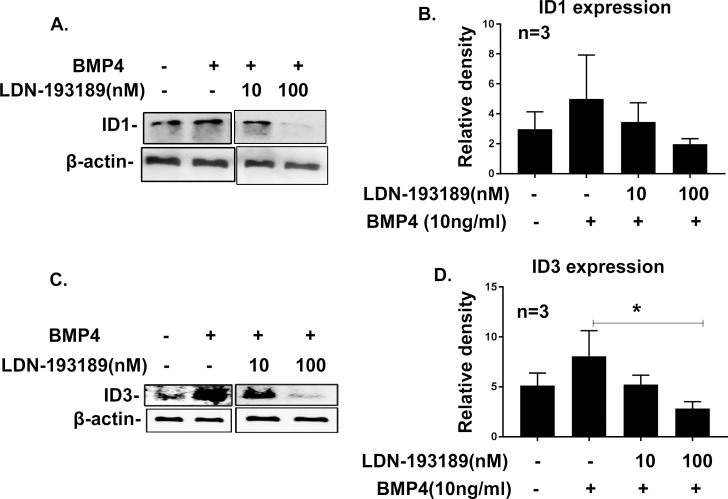
Bone morphogenic protein-RI inhibitor LDN-193189 blocks BMP4-induced ID1 and ID3 expression in TM cells. Primary TM cell strains (*n* = 3) were treated with BMP4 with or without BMPRI inhibitor LDN-193189. The inhibitor blocked ID1 and ID3 expression, confirming ID1 and ID3 are downstream targets of BMP4 signaling pathway in TM cells. Representative Western immunoblots for expression of ID1 (**A**) and ID3 (**C**) are shown. Beta-actin was used as a loading control. Densitometry analyses of ID1 (**B**) and ID3 (**D**) protein expression were normalized with corresponding β-actin expression. Expression of ID3 was significantly reduced after LDN-193189 treatment. Mean ± SEM (*n* = 3), *P < 0.05 determine using 1-way ANOVA with Dunnett test.

Having shown that BMP4 induces expression of ID1 and ID3 in TM cells, we wanted to confirm that the expression of IDs (ID1 and ID3) is dependent on BMP signaling. LDN-193189 is a BMPRI inhibitor that prevents phosphorylation of Smads1/5/8 and, therefore, inhibits the canonical BMP signaling pathway.^49,50^ We pretreated the three different serum-starved primary human TM cell strains with LDN-193189 for 6 hours and then added recombinant BMP4 (10 ng/ml) for an additional 12 hours. Protein lysates were isolated and analyzed by Western blotting. We observed inhibition of ID1 and ID3 protein expression at 100 nM and partial inhibition of IDs (ID1/ID3) at 10 nM concentration of LDN-193189 (Figs. 5A, 5C). Further the densitometry analysis demonstrated reduction of ID1 and ID3 protein expression after the LDN-193189 (100 nM) treatment (Figs. 5B, 5D). Inhibitors of DNA binding proteins 3 expression significantly decreased (*P* < 0.05) after LDN-193189 and BMP4 treatment compared to BMP4 treatment alone. This study indicated that expression of ID1 and ID3 is regulated by BMP signaling in TM cells.

### ID1 and ID3 Overexpression in TM Cells Attenuates TGF-β2 Effects

**Figure 6:**
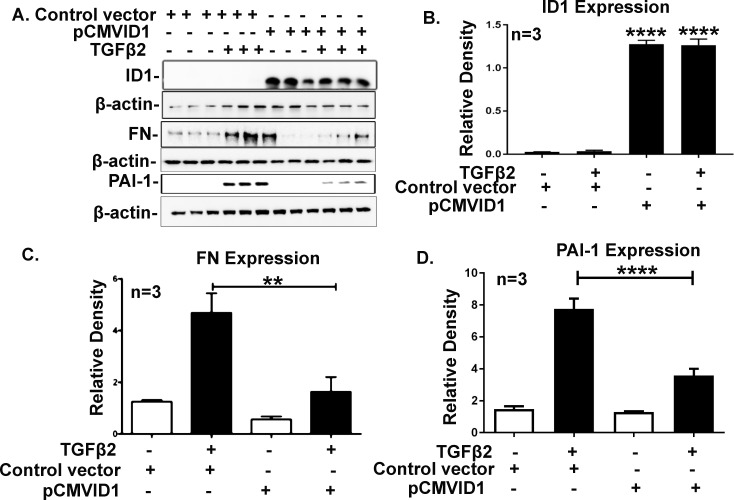
Overexpression of ID1 inhibits TGF-β2 induction of FN and PAI-1 expression. GTM3 cells transfected with the ID1 expression vector inhibited TGF-β2 (5 ng/ml) induced FN and PAI-1 expression. Western immunoblots of TM cell lysate for ID1, FN, and PAI-1 expression, after transfection of the ID1 plasmid and TGF-β2 treatment for 48 hours (**A**). Densitometric analysis of ID1 (**B**), FN (**C**), and PAI-1 (**D**) expression. Increased ID1 expression significantly reduced TGF-β2 induction of FN and PAI-1 expression. Mean ± SEM, (*n* = 3) **P < 0.01, ****P < 0.0001 determined using 1-way ANOVA with Tukey's test. Beta-actin was used as loading and normalizing control.

**Figure 7:**
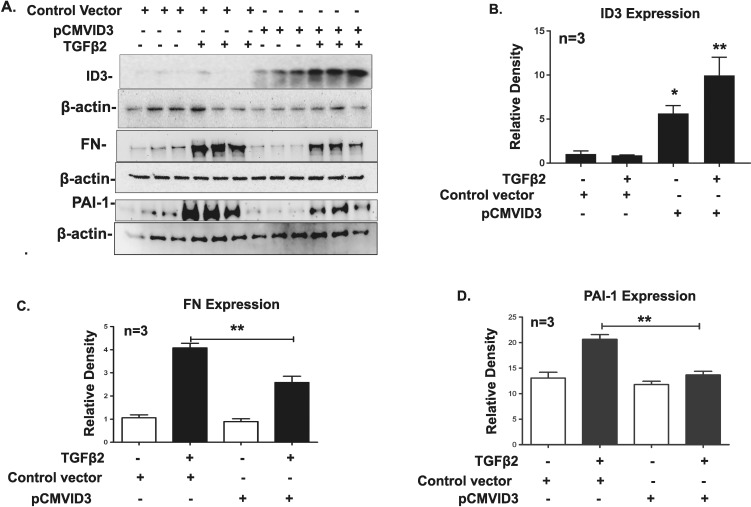
Overexpression of ID3 also inhibits TGF-β2 induction of FN and PAI-1 expression. Transformed human Glaucomatous TM (GTM3) cells were transfected with the ID3 expression vector and treated with TGF-β2 (5 ng/ml) for 48 hours. Expression of ID3, FN, and PAI-1 was observed by Western immunoblotting (**A**). Densitometric analysis of ID3 (**B**), FN, and (**C**) PAI-1 (**D**) expression was normalized with β-actin expression. Overexpression of ID3 significantly lowered FN and PAI-1 expression induced by TGF-β2. Mean ± SEM, (*n* = 3), *P < 0.04, **P < 0.01 determined using 1-way ANOVA with Tukey's test.

Transforming growth factor-β2 induces FN and PAI-1 expression along with other ECM components in primary TM cells, which can be blocked by the canonical BMP pathway.^12,23,51–53^ To determine whether overexpression of ID1 and ID3 in TM cells attenuates the TGF-β2 effects in TM cells, we transfected GTM3 cells with ID1 and ID3 expression vectors (Figs. 6A, 7A). Densitometry analyses demonstrated a significant increase in ID1 and ID3 protein expression (Figs. 6B, 7B) in ID1 and ID3 transfected cells. Cells then were treated with TGF-β2, and expression of FN and PAI-1 was studied by Western blot analysis (Figs. 6A, 7A). The FN and PAI-1 expression was normalized to β-actin loading control. Overexpression of ID1 and ID3 significantly inhibited the TGF-β2 induction of FN and PAI-1 expression (Figs. 6C, 6D, 7C, 7D; *P* < 0.01). The results demonstrated that ID1 and ID3 significantly block TGF-β2–induced FN and PAI-1 expression in TM cells.

## Discussion

**Figure 8:**
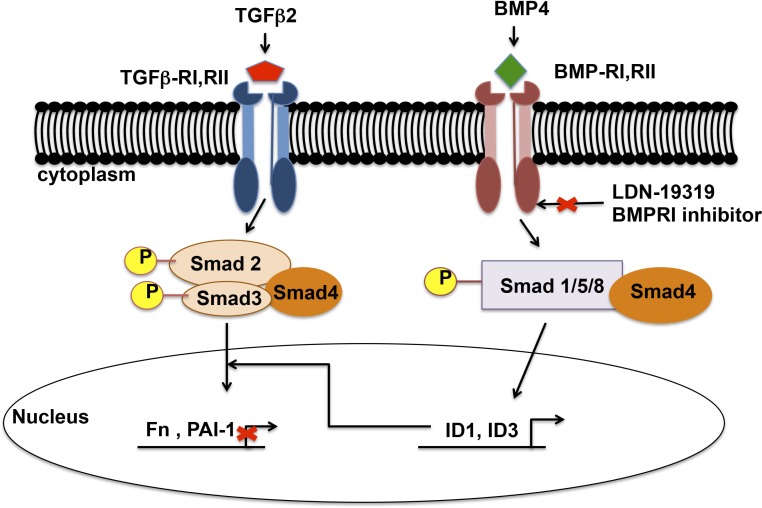
Schematic demonstrating BMP4-induced ID1 and ID3 inhibit TGF-β2–mediated FN and PAI-1 expression in TM cells. Active TGF-β2 binds to TGF-β–RI/RII to activate canonical signaling, which upregulates FN and PAI-1 expression in TM cells. Bone morphogenic protein 4 binds to BMP-RI/RII and activates Smad signaling resulting in increased ID1 and ID3 expression. Increased ID1 and ID3 proteins block the TGF-β2–induced FN and PAI-1 expression. Further, treatment of TM cells with BMPRI inhibitor LDN-193189 abolish BMP4-induced ID1 and ID3 expression, suggesting that BMP4 inhibition of TGF-β2–induced expression of FN and PAI-1 may be mediated by ID1 and ID3.

In POAG, elevated IOP remains the major risk factor for the development and progression of the disease. The ocular hypertension caused by a decreased AH outflow facility is the result of molecular and morphologic changes in the TM. Transforming growth factor-β has a critical role in maintaining the homeostasis of various tissues of the anterior segment, including regulating ECM turnover. Increased amounts of profibrotic TGF-β2 in the AH and TM of POAG patients contribute in increased production and crosslinking of ECM in TM cells leading to elevated IOP. Interestingly, BMP4 and BMP7 have been shown to block the TGF-β2 induction of ECM and ECM regulatory proteins, such as fibronectin, PAI-1, and thrombospondin-1, in TM cells. Heterozygous BMP4-deficient mice have severe anterior chamber deformities and elevated IOP, suggesting an important role of BMP4 in developing the AH outflow pathway in mice.^54,55^ Trabecular meshwork cells and tissues express BMPs (BMP2, BMP4, BMP5, BMP7) mRNA and protein as well as BMP receptors (BMPIa, BMPIb and BMPII), suggesting BMPs are essential for maintaining the homeostasis of TM tissue.^22^ Fuchshofer et al.^24^ demonstrated that BMP7 blocked the TGF-β2 effects in TM cells by upregulation of inhibitory Smad7. However, the blocking mechanism of BMP4 on profibrotic TGF-β2 signaling in TM cells remained unknown. In our study, we showed that ID1 and ID3 are key downstream regulators of the BMP4 signaling pathway, and expression of ID1 and ID3 proteins are BMP pathway–dependent, inhibiting TGF-β2–induced FN and PAI-1 expression in human TM cells. These results further suggested that BMP4 regulated inhibition of TGF-β2–induced FN and PAI-1 may be mediated by ID1 and ID3 (Fig. 8).

Inhibitors of DNA binding proteins (ID1–ID4) are dominant negative transcriptional regulators expressed in different tissues, including lungs, kidney, cardiovascular tissue, reproductive organs, and neuronal tissues.^56–58^ Inhibitors of DNA binding proteins have critical roles in early embryonic development and demonstrate overlapping function during cell cycle progression.^31,32,59^ However, IDs promote cell proliferation and regulate cell differentiation depending on cell type and cellular function. For example, IDs inhibit cell differentiation in neural progenitor cells to maintain a neural cell population, while promoting natural killer (NK) cell differentiation.^58,60^ Hence, expression and function of IDs are cell- and tissue-specific. Knockdown of both *ID1* and *ID3* genes is lethal for mouse embryos during development due to impaired angiogenesis and neurogenesis.^25,61^ During development, the expression of ID1, ID2, and ID3 are predominant in neural crest and neural cells.^31,58^ Expression of ID4 has distinctively selective functions in neuronal proliferation and differentiation.^30,62,63^ Recent reports suggest that IDs have important roles in retinal development, bipolar cell lineage commitment, and differentiation and fibrotic corneal disease.^33,64,65^ However, very little was known about ID expression and their role in human TM cells. To our knowledge, for first time we demonstrated basal expression of ID1, ID2, and ID3 in primary TM cells. Our published microarray data of human TM tissues suggested expression of all four IDs (ID1–4).^66^ However, we failed to detect the expression of ID4 in our primary TM cell cultures. In our unpublished data in GTM3 cells, the knockdown of ID1 by siRNA upregulated ID3 expression and vice versa. In addition to our observation, several other studies have demonstrated that expression of ID1 and ID3 is highly correlated.^45,67^ Heterozygous ID1/ID3 knockout mice demonstrated suppression of BMP-induced bone formation.^68^ Additionally, reports show that ID1 is involved in regulation of fibrosis by inhibiting TGF-β–induced FN and PAI-1 in various cells types. Therefore, we selected ID1 and ID3 to investigate their roles in the TM cell and BMP4 signaling pathway. We demonstrated the expression of ID2 in primary human TM cell strains, and its role in TM cells should be studied further.

Bone morphogenetic proteins (BMP2, BMP4, BMP5) are known as potent inducers of ID1 and ID3 expression in various other tissues. Bone morphogenic protein 4 induces expression of IDs in different cells, including mesenchymal cells, endothelial cells, and mesangial cells.^13,63,69–71^ Bone morphogenic protein 4 binds to BMPRI and RII, which phosphorylates Smads 1/5/8, resulting in activation of the BMP response element on *ID* genes, thereby upregulating their expression.^27,28^ In our studies, we reported significant upregulation of ID1 and ID3 mRNA expression after BMP4 treatment in the TM cells. Several groups have reported that BMP induction of ID1 and ID3 mRNA expression is biphasic and cell-dependent, which peaks from 30 minutes to 48 hours depending on the cell type.^35,72,73^ Similar to these previous findings, our study indicates a rapid induction of ID1 and ID3 mRNA within 1hr after BMP4 treatment. While ID1 mRNA expression significantly increased at 2, 12, and 24 hours, ID3 mRNA expression was significantly increased at 2 and 24 hours after BMP4 treatment in different primary TM cell cultures.

While TGF-β2 (5 ng/ml) treatment of primary human TM cell strains (*n* = 5) exhibited no induction of ID2 and ID3 mRNA expression, ID1 mRNA expression showed high variability among cell strains (Supplementary Fig. S1). Further, we also reported significant induction of ID1 protein from 2 to 24 hours and ID3 protein induction from 1 to 48 hours in BMP4-treated primary human TM cells. The basic understanding of ID regulation of gene expression is through repression of E-box promoter regions, as IDs bind to E-box promoter regulators, especially E2A gene products (E12 and E47).^31^ However, current advances have demonstrated diverse roles of ID proteins. Inhibitors of DNA binding proteins 1 may regulate antifibrotic effects independent of E-box promoter regulators.^34,48,74^ Apart from their role of inhibiting and regulating gene transcription, ID1 also binds to estrogen receptor β (ERβ) and inhibits breast cancer cell proliferation.^46^ Due to their varied cellular functions in different cell types, ID1 and ID3 proteins are localized in the nucleus as well as in the cytoplasm of cells.^48,75^ Our studies demonstrated a similar pattern of expression of ID1 and ID3 proteins in the primary human TM cells. However, after BMP4 treatment, expression of ID1 protein increased in cytoplasm as well as nucleus, while ID3 expression increased in nucleus of primary human TM cells. Therefore, these studies suggested BMP4 significantly induces ID1 and ID3 mRNA and protein expression and controls their localization in primary TM cells.

To further confirm our hypothesis that ID1 and ID3 expression is BMP4 pathway–dependent, we pretreated primary human TM cells with BMPRI inhibitor LDN193189. This inhibitor inhibits BMPRI activity and, therefore, inhibits downstream signaling of the canonical BMP4 pathway by preventing phosphorylation of Smads 1/5/8. The complete inhibition of pSmads 1/5/8 at higher concentrations of LDN193189 has been reported.^49,50^ In our study, we demonstrated pretreatment with LDN193189 similarly reduces BMP4 induction of ID1 and ID3 (*P* < 0.05) protein expression. However, we did not observe statistically significant reduction of ID1 protein expression compared to BMP4-treated positive controls due to the variable response in primary human TM cell strains. However, we observed significant increase of ID1 protein expression by BMP4 earlier (Figs. 3A, 3B). This study suggested that expression of ID1 and ID3 in TM cells is BMP pathway–dependent.

Transforming growth factor-β2 increases the expression of various ECM components such as FN, collagen IV, and thrombospondin-1, thereby increasing ECM levels in the TM. Transforming growth factor-β2 also increases expression of the ECM crosslinking enzymes LOX, LOXL1-4, TGM2, and BMP1 as well as inhibitors of ECM degradation by increasing expression of PAI-1 and TIMP. This would decrease ECM degradation and increase outflow resistance in the TM.^18,52,76–78^ Transforming growth factor-β2 in human and porcine anterior segment perfusion organ cultures and in rodent eyes increases IOP and decreases outflow facility as a result of increased in ECM deposition.^12,79,80^ Transforming growth factor-β2–induced PAI-1 expression in TM cells inhibits the activation of proteolytic system (matrix metalloproteinases [MMPs]) resulting in reduction of ECM turnover.^12^ Treating TM cells with recombinant PAI-1 also increases FN expression.^12^ Similarly, increased expression and secretion of FN isoform ED-A leads to formation of insoluble extracellular fibrils.^18,44^ These increased FN extracellular fibrils bind integrins, which increase actin stress fiber formation and may lead to IOP elevation.^81^ Interestingly, we demonstrated IDs (ID1/ID3) can significantly downregulate the expression of FN and PAI-1 induced by TGF-β2 in human TM cells. Further studies in dermal fibroblast demonstrate that ID1 inhibits TGF-β–induced fibrosis through reducing the expression of phosphorylated Smad2 and Smad3.^37^ Also, ID1 is known to bind to caveolin-1 and may mediate internalization of the TGF-βRI receptor, as seen in alveolar epithelial cells.^81^ The precise mechanism by which ID1 and ID3 inhibit FN and PAI-1 in TM cells requires further investigation.

In summary, to our knowledge this is the first report to demonstrate expression of ID proteins and their inhibitory effects on TGF-β2–induced FN and PAI-1 expression in primary TM cells. Our study showed basal expression of ID1, ID2, and ID3 in primary human TM cells. Primary human TM cells treated with BMP4 significantly induce ID1 and ID3 mRNA expression. Furthermore, BMP4 induces early expression of ID1 and ID3 mRNA and proteins. Expression of ID1 and ID3 in primary human TM cells is BMP pathway–dependent. Expression of ID1 and ID3 can suppress the TGF-β2 effects in TM cells. Therefore, this study suggested that BMP4 blocking of TGF-β2–induced FN and PAI-1 appears to be mediated by ID1 and ID3. Our study also suggested that these novel regulators can inhibit the profibrotic TGF-β2 signaling pathway and may therapeutically prevent disease progression in the glaucomatous TM.

## Supplementary Material

Supplement 1
