# Supplementary material for: Role of ID Proteins in BMP4 Inhibition of Profibrotic Effects of TGF-β2 in Human TM Cells
Source: Invest Ophthalmol Vis Sci. 2017 Feb;58(2):849–59. doi: 10.1167/iovs.16-20472 (PMC5295782; doi:10.1167/iovs.16-20472)
Supplement: Supplement 1 [file iovs-58-01-13_s01.pdf]

Supplementary Figure 1

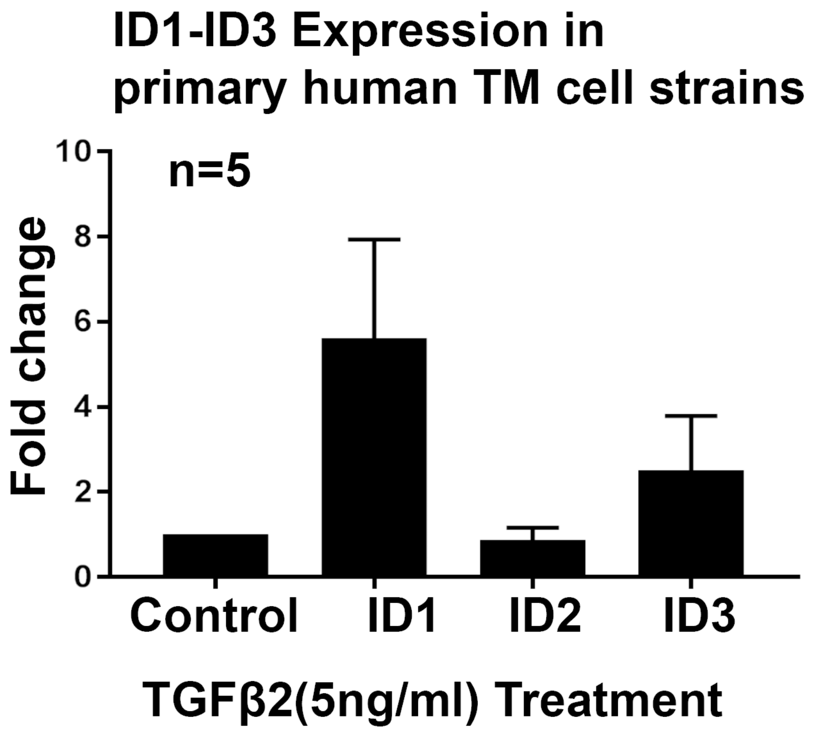

Supplemenatry Figure 2.

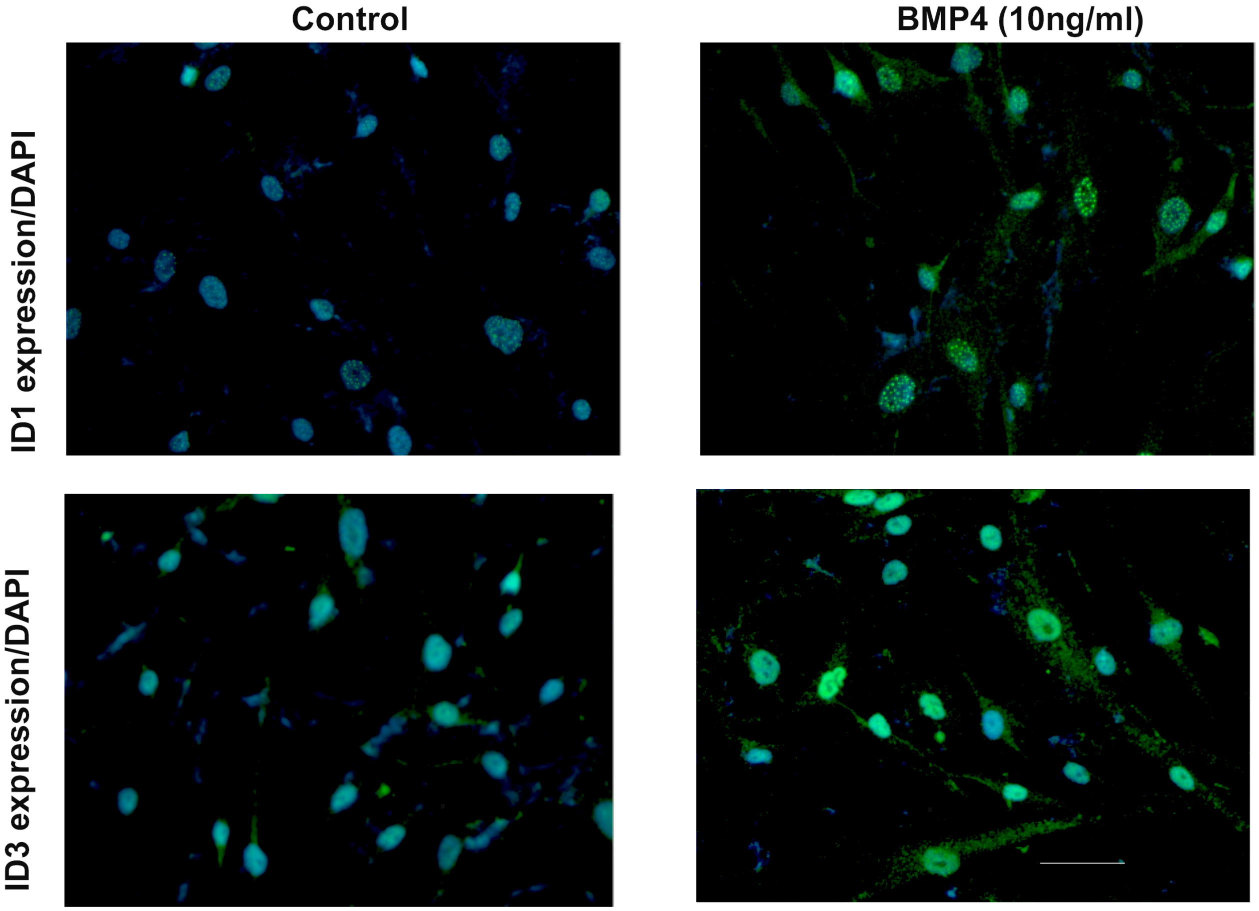

Supplementary Figure Legends

Supplementary Figure 1. Effect of TGFβ2 on the expression of ID1, ID2, and ID3 mRNA in TM cells. Five human primary TM cell strains were treated with TGFβ2 (5ng/ml), and mRNA was isolated post TGFβ2 treatment. There was no significant change in the expression of ID2 and ID3. However, ID1 showed an apparent increase in expression, but due to the high variability observed among the cell strains, this was not statistically significant.

Supplementary Figure 2. ID1 and ID3 expression increased in the nucleus and cytoplasm post BMP4 (10ng/ml) treatment (12 hrs). Higher magnified 400X images suggest increased nucleus
